# Large adrenocortical adenoma with malignant features on imaging: A case report

**DOI:** 10.1016/j.eucr.2021.101968

**Published:** 2021-12-06

**Authors:** Shin Koike, Shotaro Nakanishi, Sunao Nohara, Hirofumi Miyahira, Tomoko Tamaki, Seiichi Saito

**Affiliations:** a Department of Urology, University of the Ryukyus, Okinawa, 9030215, Japan; b Department of Diagnostic Pathology, University of the Ryukyus, Okinawa, 9030215, Japan

**Keywords:** Adrenocortical adenoma, Adrenal incidentaloma, Adrenal mass, Adrenocortical carcinoma, CT, computed tomography, MRI, magnetic resonance imaging, ^18^F-FDG PET/CT, F-18 fluorodeoxyglucose positron emission tomography/computed tomography, SUVmax, maximum standardized uptake value

## Abstract

Large adrenocortical adenomas have rarely been reported. We describe a case of a 26-year-old man who underwent an adrenalectomy for a large adrenocortical adenoma (8.6 × 7.7 cm). Although the lesion had typical malignant features on imaging, histopathological examination revealed an adrenocortical adenoma. This highlights that imaging alone may not be able to distinguish adrenocortical carcinomas from adrenal masses. In most cases, a resection should be performed for early diagnosis and management of large adrenal masses with malignant features on imaging. To our knowledge, this is the first report of a large adrenocortical adenoma diagnosed with multiple imaging investigations.

## Highlights

• A patient presented with abdominal and left flank pain.
• We observed an ≥8-cm lesion that appeared to be malignant on imaging.
• Histologically, the lesion was diagnosed as an adrenocortical adenoma.
• Adrenocortical adenomas with malignant imaging features must be resected.
• This will allow their early diagnosis and treatment.

## 1 Introduction

Adrenal masses are often classified as incidentalomas, because most are detected during the work-up for non-adrenal diseases. Most large adrenal masses are potentially malignant; benign large adrenal tumors have rarely been reported.^1^^,^^2^ Here, we report a rare case of a large adrenocortical adenoma presenting with some malignant radiologic features.

## 2 Case presentation

A 26-year-old man visited a general hospital for abdominal and left flank pain. Contrast-enhanced computed tomography (CT) confirmed the diagnosis of appendicitis. However, a left adrenal mass was incidentally identified, which prompted referral to our hospital for further investigation and treatment.

**Fig. 1:**
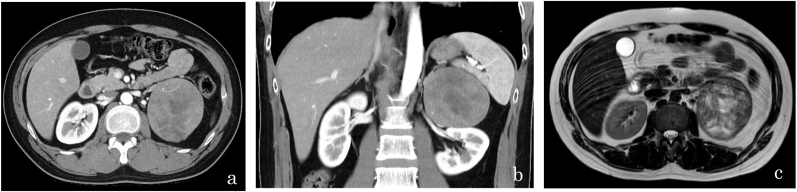
Radiologic findings. (a, b) Contrast-enhanced computed tomography revealing an 8.6 × 7.7-cm heterogeneously enhancing left suprarenal mass with increased vascularity. (c) T2-weighted magnetic resonance imaging showing a hyperintense left adrenal mass.

Contrast-enhanced CT revealed an 8.6 × 7.7-cm, heterogeneously enhancing mass in the left suprarenal region with increased vascularity (Fig. 1a and b). Similarly, T2-weighted and diffusion-weighted magnetic resonance imaging (MRI) revealed a hyperintense suprarenal mass (Fig. 1c).

**Fig. 2:**
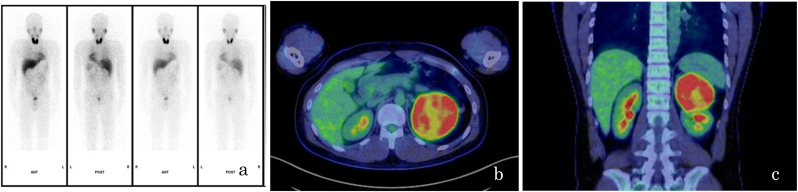
Imaging findings. (a) ^123^I-meta-iodobenzylguanidine scintigraphy showing no signs of a pheochromocytoma. (b, c) F-18 fluorodeoxyglucose positron emission tomography/computed tomography revealing strong uptake in the left adrenal region with a maximum standardized uptake value of 21.39.

All hormonal studies were negative. ^123^I-meta-iodobenzylguanidinescintigraphy showed no signs of a pheochromocytoma (Fig. 2a). F-18 fluorodeoxyglucose positron emission tomography/CT (^18^F-FDG PET/CT) showed a strong uptake in the lesion, with a maximum standardized uptake value (SUVmax) of 21.39 (Fig. 2b and c).

**Fig. 3:**
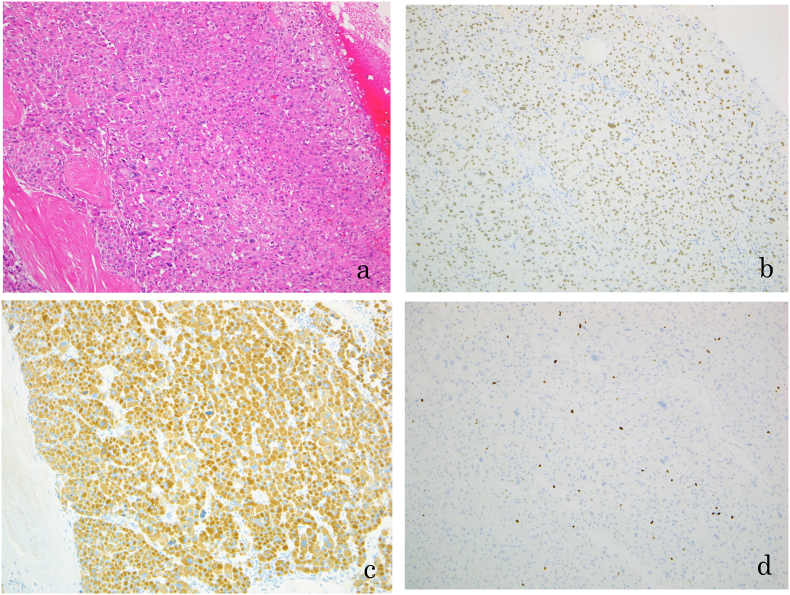
Histopathological findings. (a) H&E staining (×100): section of the adrenocortical adenoma showing solid sheets and nests of acidophilic cells with large and small nuclei, Immunohistochemistry of the adrenocortical adenoma; (b) SF-1 (×100): positive; (c) alpha-inhibin (×100): positive; (d) Ki-67 (×100): low Ki-67-labeling proliferating index (1%–2%). H&E, Hematoxylin and eosin; SF-1, Steroidogenic factor 1.

A left open adrenalectomy was performed. Intraoperatively, the tumor had a relatively distinct margin with mild adhesions between the psoas muscle. The histopathologic findings were consistent with the diagnosis of an adrenocortical adenoma as the tumor did not meet any of the Weiss criteria (Fig. 3a, b, c, d). The patient's postoperative course was uneventful with resolution of the flank pain.

## 3 Discussion

Incidentalomas are lesions that are detected during routine imaging investigations for other diseases. With the increasing use of radiologic techniques, the incidence of incidentalomas is increasing continuously.^3^ The size of the mass is correlated to its potential for malignancy.^1^ According to Mohamed et al., up to 70% of adrenal masses ≥4 cm were associated with malignancies.^1^ Among masses greater than 6 cm, 85% were malignant.^1^

Typical imaging features and sizes of adrenal masses are variable. In the present case, the tumor had typical characteristics of an adrenocortical carcinoma (i.e., heterogeneous on CT, hyperintense on T2-weighted MRI, and size ≥4 cm).^2^Furthermore, ^18^F-FDG PET/CT showed a strong uptake in the left adrenal region (SUVmax = 21.39). Although these features were highly suggestive of malignancy, the tumor did not fulfill the Weiss criteria. According to recent studies, ^18^F-FDGPET with or without CT had high sensitivity (91%) and specificity (91%); the positive and negative likelihood ratios were 9.9 and 0.09, respectively.^4^ Despite this diagnostic accuracy, the utility of these techniques for the characterization of adrenal masses remains limited. These modalities are not highly recommended for patients without a known malignant lesion.^5^

Resection is recommended for adrenal masses ≥4 cm. In this case, a left adrenalectomy was performed. Considering the lesion's typical malignant features, we expected a pathological diagnosis of an adrenocortical carcinoma. However, fortunately, the histopathologic findings were consistent with an adrenocortical adenoma.

## 4 Conclusion

Herein, we report a case of a large adrenocortical adenoma that radiographically presented as a malignancy. Ours is the first report of a large adrenocortical adenoma diagnosed with multiple imaging investigations. Our findings suggest that imaging may not adequately differentiate between adrenocortical carcinomas and incidentalomas. Adrenocortical adenomas presenting with malignant imaging features must be resected for early diagnosis and treatment.

## Author contributions

**Shin Koike, Shotaro Nakanishi**; Conceptualization, Investigation, Writing - Original draft preparation, Writing - Reviewing and Editing.

**Sunao Nohara, Hiroshi Miyahira, and Tomoko Tamaki**: Writing - Original draft preparation.

**Seiichi Saito**: Supervision.

## Consent

Written informed consent was obtained for the publication of this case report.

## Declaration of interest

None.

## Funding

This research did not receive any specific grant from funding agencies in the public, commercial, or not-for-profit sectors.
